# Environmental and Geotechnical Assessment of the Steel Slags as a Material for Road Structure

**DOI:** 10.3390/ma8084857

**Published:** 2015-07-30

**Authors:** Wojciech Sas, Andrzej Głuchowski, Maja Radziemska, Justyna Dzięcioł, Alojzy Szymański

**Affiliations:** 1 Water Centre Laboratory, Faculty of Civil and Environmental Engineering, Warsaw University of Life Sciences, 02-787 Warsaw, Poland; E-Mail: justyna_dzieciol@sggw.pl; 2 Department of Geotechnical Engineering, Faculty of Civil and Environmental Engineering, Warsaw University of Life Sciences, 02-787 Warsaw, Poland; E-Mails: andrzej_gluchowski@sggw.pl (A.G.); alojzy_szymanski@sggw.pl (A.S.); 3 Department of Environmental Improvement, Faculty of Civil and Environmental Engineering, Warsaw University of Life Sciences, 02-787 Warsaw, Poland; E-Mail: maja_radziemska@sggw.pl

**Keywords:** steel slag, road structure, recycling, California bearing ratio (CBR), static load testing

## Abstract

Slags are the final solid wastes from the steel industry. Their production from waste and associated materials is a proper implementation of the basic objectives and principles of the waste management. This study aims to investigate the chemical and selected significant geotechnical parameters of steel slag as the alternative materials used in road construction. These investigations are strongly desired for successful application in engineering. Young’s modules *E*, and resilient modules *M*_r_ showed that their values corresponding with requirements for subbase (principal or auxiliary) and riding surface as well. Tested mechanical properties were conducted in soaked and un-soaked (optimal moisture content) conditions. The designated high content of chromium and zinc are strongly associated with the internal crystal structure of steel slag. The results do not lead to threats when they are applied in roads’ structures. Mechanical characterization was obtained by performing California bearing ratio (CBR) tests for steel slag in fixed compaction and moisture content conditions. Moreover, cyclic loading of steel slag was conducted with the application of cyclic California bearing ratio (cCBR) apparatus to characterization of this material as a controlled low-strength material. Finally, field studies that consist of static load plate VSS tests were presented.

## 1. Introduction

Construction engineering, as well as the building materials industry, makes a significant contribution to the production of industrial wastes and environmental pollution. On the flip side, this industry utilizes a large amount of waste, using the industrial waste as raw materials or ingredients of building materials, an example of which can be steel slags [1]. These are the final waste products from the steel industry and make up a proportion of approximately 15% by mass of the steel output [2]. Steel slag is produced from Basic Oxygen Furnace and Electric Arc Furnace in steel making and the main chemical compositions include CaO, SiO_2_, Al_2_O_3_, Fe_2_O_3_, MgO and FeO [3,4,5]. 

Huawei [6] reports that the production of each of these three tons of stainless steel will create one tonne of waste. According to European Slag Association (EUROSLAG), in Europe, a production of steelmaking slag is 21.8 Mt in 2010 [7]. The refining process of the so-called secondary steel making operation produces stainless steel reducing slag (SSRS). SSRS includes argon oxygen decarburization and ladle metallurgy slag [8]. When SSRS compares with ground granulated blast furnace slag (GGBFS) from iron making, steel slag contains toxic ingredients such as nickel, cadmium, chromium and strontium. These compounds could be harmful not only for environment but also for human health [9,10]. Steel slag recycling issues have gained importance in research areas and environmentally friendly processes.

Artificial and recycled aggregates are alternative building materials [11,12]. Their production from waste and associated materials is a proper implementation of the basic objectives and principles of waste management such as: reduction of the amount of waste and its negative impact on the environment, recovery of materials, disposal of waste and production of artificial aggregates. The application of such materials requires preliminary strength property estimation. These properties depend on many variants, which were summarized in ACI 229R-99 [13]. Many materials, such as plastic-soil elements and slurry material, were proposed as controlled-low strength materials (CLSM). CLSM could consist of many various materials—for example, recycled concrete aggregates that could be deposited on construction site in unbound or bound form, with addition of lime in the last case [14,15,16,17]. Many low strength clayey soils could also be a base for CLSM [18,19]. CLSM can compound from various chemically active substances but also can contain another materials which improve physical properties as rubber waste from tires to decrease the weight of CLSM [20].

In the case of steel slag, this material could be deposited in its raw state as a subgrade or subbase material. The application of steel slag in asphalt concrete also gives good performance [21,22]; however, the use of steel slag as a construction material has been a problem, because free CaO abundant in steel slag in contact with water transforms into Ca(OH)_2_ [23]. Moreover, steel slag could be subjected to various treatments. The activation of hydraulic properties during crushing is one of them [24]. A large amount of steel slag is employed, as was mentioned above, to road construction as an aggregate in concrete production or fertilizer production [25] and after relevant treatments as a hydraulic binder [26,27], to replace river sand for preparing the waterproof mortar [28]. Steel slag could be used as a source of reactive elements for the purpose of CO_2_ isolation [5].

CLSMs under their own weight consolidate themselves. This property allows the replacement of natural soil by those materials [13]. An important feature of CLSM as opposed to virgin soils, is that, after the compaction and hardening process, CLSMs do not stop to settle. Hardening obtained by controlled-low strength materials (CLMS) is greatly dependent on the quantity of cementitious material which it contains. Hardening time can be as short as one hour but usually takes three to five hours to reach proper bearing capacity conditions. Requirements for such materials as CLSM are to achieve low compressive strength of 0.5–2.0 MPa for possible re-excavation in the future [29]. ACI-229R requirements for compressive strength are of 8.3 MPa or less, for steel slags (7.23 MPa), this requirement is fulfilled [30]. During CBR tests, steel slag also exhibited the abovementioned properties and is discussed in this paper. 

The utilization method of steel slag is closely related to the chemical and physical characteristics and should be given a priority from environmental considerations. Nowadays, almost 100% of steel slag is utilised in many areas, such as cement production, raw material for brick production, landfill daily cover, road construction, civil engineering work, filtering media for waste water treatment and fertilizer production [31,32]. 

Nevertheless, for road construction, the possibility of using unbound material with the potential to a self-cementing feature in time after the mechanical stabilization is desired. Possible settlement due to consolidation and repeated loading could be limited by a volume expansion of steel slag when it reacts with free lime or another alkali compounds [33]. Steel slag could be also milled to powder form which exhibits cementitious properties [9,34,35] and after compaction of unbound material could be filled with slag powder to create a mortar.

In recent years, there has been a significant increase of the usage of these aggregates in road construction in Poland. Very good quality of road surface, together with the pro-environmental and economic reasons, support their wider utilization. The technical requirements for unbound aggregates in the road construction in Poland for natural aggregates are presented in the appropriate document [36]. 

Mechanical characterization of materials such as steel slag are indicated in road engineering by performing the CBR test. The CBR values are key factors of estimating the roadbed thickness. The evaluated strength of subgrade filling give the indices of different highway grade and different road base requirements. This procedure is an element of empirical road design. The right choice of materials, which was characterized by this value, ensure the quality of road construction which nowadays play huge role [37].

The aim of this study was to determine the chemical and selected significant geotechnical parameters of steel slag as the alternative materials used in roads construction, as well as to confirm their environmental and technical quality.

## 2. Materials and Methods

### 2.1. Material and Sample Preparation

In order to evaluate the chemical composition and obtain geotechnical parameters, the steel slag samples were collected from a metallurgical landfill, which provided the examined aggregate to improve the municipal low frequented village road. The test site was a real proving ground, the municipal road with the asphalt surface. It was the main road of the L class, traffic categories KR5 in polish standards and by the ARRB AR354 [38] is represented by class 50, which is the local throughout route. The subbase of this road was constructed from steel slag. Steel slag was cut off by construction of the drainage layer under the subbase to prevent capillary actions. Over the subbase, an impermeable asphalt layer was structured. Such construction leads to averting any outflows and inflows of water in a subbase layer. 

The commercial aggregate samples had the size fraction of: 0–10 mm; 0–31 mm, and 0–63 mm. After compaction of a steel slag layer, field tests were performed. Four static load plate VSS tests in distant points were conducted until the first loading caused 0.8 mm of surface displacement. During these tests, stress and displacement were noted.

### 2.2. Laboratory and Chemical Analysis

Based on averaging collected material, melted below 80 μm by using a centrifugal mill (Retsch ZM100, Haan, Germany) and powdered samples were prepared. The chemical composition was determined by wavelength dispersive X-ray fluorescence (XRF) spectrometry analysis on Philips PW 2400 (Eindhoven, The Netherlands), quantified using the Super Q software. The heavy metal content was analyzed with inductively coupled plasma optical emission spectrometry (ICP-OES, Varian 720-ES, Mulgrave, Australia). The operating conditions were: argon gas used as plasma gas flow at the rate of 15.0 L·min^−1^, auxiliary gas flow rate 1.50 L·min^−1^, nebulizer gas flow rate 0.75 L·min^−1^. The room temperature was fixed at 25 °C for the analyses. Analyses were performed three times. Samples were digested with nitric acid (Merck, Darmstadt, Germany, 69% m/v) on a microwave oven (Milestone, Italy). All reagents were of analytical reagent grade unless otherwise stated. Stock solutions of metals (1000 mg/L) were prepared from their nitrate salts. Ultra-pure (UP) water (Millipore System, Bedford, MA, USA) 0.055 μS·cm^−1^ resistivity was used for preparing the solutions and dilutions for all dilutions. 

### 2.3. Geotechnical Laboratory Analysis

Geotechnical laboratory analysis consisted of a number of bearing capacity tests according to PN-S02205: 1988 [39] so-called CBR tests [40]. The test relates the bearing capacity of the material in CBR test conditions to that of a standard crushed gravel represented as % of standard material bearing capacity. Representative specimens were prepared from large samples of slag material, with respect to Proctor’s method, preliminary tests lead to estimate optimal moisture content equal 14.9% at dry density equal 2.02 g/cm^3^ . Four special mixture aggregates: 2–10 mm; 2–25 mm; 2–10 mm and 2–25 mm with 30% addition fraction 1–2 mm were tested. The reason of the addition of 1–2 mm fractions was to simulate real field conditions connected with the seal of surface subgrade and to prevent them from loose contact between plungers and specimens in laboratory tests. Each compound was studied ten times.

### 2.4. Cyclic Loading Tests

Repeated loading on soil samples prepared in CBR mould was performed with the use of cyclic CBR test (cCBR) procedure. cCBR method was based on a common CBR test. The main idea behind using this equipment came from its popularity. The long existence of the CBR method and its usefulness in road design, resulted in its worldwide spread. 

By using CBR, mould and repeated loading apparatus, the cCBR test method was established. The main principle of this test approach is to use standard CBR test procedures as a reference in order to study the later cyclic loading stage. 

As was mentioned above, the first step is standard CBR test loading to 2.54 mm. After reaching the desired displacement with the use of a plunger, the unloading procedure was attempted, with the use of up to 10% of force obtained at 2.54 mm. Loading and unloading is treated as the first cycle of the cCBR test. The next cycles are determined by the maximal and minimal force from the first loading. The test was carried out with standard 1.27 mm/min. velocity. The number of cycles is determined by the percentage of plastic strain in one cycle. cCBR method assumes that the test can be stopped, when 1% or less of plastic displacement in one cycle will occur. The amount of the cycles to obtain this condition usually oscillates around 50 [41,42].

## 3. Results and Discussion

### 3.1. Chemical and Mineralogical Characterisation of the Slag

The chemical composition of steel slag varies with the furnace type, steel grades and pre-treatment method. The chemical composition, by XRF, of the steel slag is given in Table 1. 

**Table 1:** Chemical composition of steel slag samples, mass percent (%).

CaO free	SiO_2_	TiO_2_	Al_2_O_3_	Fe_2_O_3_	MnO	MgO free	Na_2_O	K_2_O	P_2_O_5_	SO_3_	Cl	F
27.46	16.69	0.43	6.64	33.82	3.87	6.68	0.68	0.10	0.30	0.45	0.003	2.89

The composition of steel slag is variable and may depend on, among others, the size of fraction. The main primary solid phases consist of a Fe_2_O_3_. The main mineral compounds are metallic iron (dicalciumsilicate, dicalciumferrite) [4]. The two major mineral phases present in the steel slag samples were hematite Fe_2_O_3_ and lime (CaO), content of ~34 wt % and ~27 wt %, respectively. Other minor phases identified were quartz (SiO_2_) and Al_2_O_3_. The content of CaO, MgO, SiO_2_ and Al_2_O_3_ that may principally substitute raw materials for cement production [43,44]. Because of the disintegration process, the residue values of free lime (CaO), are most harmful for unbound steel slag composition. Concentrations of lime (CaO) are dominant in steel slag [45]. The content of free CaO and free MgO is the most important factor for the disposal of slag and their use in the building industry because of their durability volume. Steel slag with CaO content above 50% can be used as sinter ore fluxing agent, partially replacing the commercial lime. 

In contact with water, these minerals react with hydroxide, depending on the level of free lime and free MgO reaction causes an increase in the volume of slag, which is mainly linked to the breakdown of the particles and a decrease in the strength. The stability of the volume is a key criterion by using slag as a construction material. The analyzed material was characterized by a relatively low content of free CaO and MgO, so that significant changes in its volume as well as a decrease of its strength in time can be excluded. Experience in Germany has found that steel slags with a free lime content up to 4% in asphaltic layers and up to 7% may be used in unbound layers [4]. Some steel slags contain a higher amount of P_2_O_5_; this may affect the direct recycling of the steel slags to the iron and steel making process. Steel slag was identified as a material which causes 10% higher P retention capacity in columns for phosphorus removal than columns without steel slag [46]. This component might lead to decomposition of C_3_S, which reduced activity of steel slag. The silica (SiO_2_) content in slag samples was 16.69%, the Al_2_O_3_ and MnO contents are in 6.64% and 3.87%, respectively. The silicate glass at the level 42.38–78.23 wt % SiO_2_ and 7.48–38.87 wt % Al_2_O_3_ were found in slag from the former Hegeler Zn-smelting facility in Illinois (USA) [47]. Puziewicz *et al.* [48] reported for a Zn-smelter waste dump in Upper Silesia (Poland) having 12.43–41.27 wt % SiO_2_, 3.38–20.69 wt % Al_2_O_3_, 9.86–29.68 wt % Fe_2_O_3_ and 4.97–23.53 wt % CaO.

Heavy metal toxicity and mobility in the natural environment depends on their chemical speciation. In steel, slags are present in relatively high values [49]. 

Steel slag contains trace amounts of elements potentially mobile and toxic to the environment [50]. To get information about the effect on the soil and ground water, it is interesting to know that the concentrations of those environmentally relevant components can be leached out. Some steel slags contain higher amounts of toxic metals, such as chromium, nickel, manganese, vanadium and molybdenum [51]. Lottermoser [52] identified in slag from Río Tinto (Spain) contain elevated concentrations of potentially toxic trace elements such as As, Cd, Co, Pb, Sb and Zn.

Steel slag from analysing samples also consists of several different types of heavy metals in various concentrations. The heavy metal composition of the steel slag samples is given in Table 2. Results of the analysis of samples confirm a high content of chromium and zinc. The analysis of the chemical composition of steel slag shows the content of Cr at the level of 2 915 mg·kg^−1^. Chromium exists in slags as magnesiochromite (MgO·Cr_2_O_3_) or solid chromium oxide (Cr_2_O_3_). In contact with water the tri- and hexavalent chromium (highly soluble in water) can be released from the slag by means of a leaching process [53]. The hexavalent cation can be produced by oxidation with atmospheric oxygen and contact with CaO. Leaching of Cr increases as soon as the iron (Fe^2+^) is oxidized into Fe^3+^. Zinc has a high affinity for mineral colloids, characterized by a high mobility in the soil and the high bioavailability of the plants due to the rate of dissolution of the compounds in which they occur, in particular in an acidic environment [54,55]. In the tested sample of slag, zinc was the second in terms of the content of the element (1 084 mg·kg^−1^). This value is similar in composition to Zn-rich slags from Poland with 2600–27,200 mg·kg^−1^ [45] and 212 to 14,900 mg·kg^−1^ in slag from the former Hegeler Zn-smelting facility in Illinois (USA) [47]. In sandy soils, zinc can be toxic at concentrations of 6.9–12.8 g·kg^−1^ of soil, in clay soils it is revealed at the higher concentrations of 16.2–21.5 g·kg^−1^ soil. In the presented study, concentrations of the barium in steel slag samples were 380 mg·kg^−1^. According to Piatak and Seal [47], the content of Ba in slag was between 788 and 1170 mg·kg^−1^. The tested steel slags were characterized by less than half as much Cu as niobum, lead, and nickel. Studies of authors [47] examined the As contained in slags from Hegeler and the value of As was 1–45 mg·kg^−1^; in samples presented in this study, the concentration of arsenium value was 10 mg·kg^−1^. Other elements that may pose a potential risk of soil degradation occur in samples of slag at levels exceeding the limit values for soil category A: cobalt (Co) and nickel (Ni); exceeding the limit values for soil category A, but allowing free use of the soil category B and C: molibdenium (Mo), lead (Pb) and cadmium (Cd). To limit the effects of toxic elements, leaching from slag should be minimized to reduce long term leaching and minimized contact with water e.g., cut of water inflow and outflow in layers constructed with use of steel slag to prevent toxic effects of chromium from slag to environment.

In the tested sample of slag, zinc was second in terms of the content of the element. Other elements that may pose a potential risk of soil degradation, occur in samples of slag at levels exceeding the limit values for soil category A: arsenium (As), cobalt (Co) and nickel (Ni); exceeding the limit values for soil category A, but allowing free use of the soils category B and C: cupper (Cu), molibdenium (Mo), lead (Pb) and cadmium (Cd). 

**Table 2:** Total composition of the steel slag samples, mg·kg^−1^.

Element	Value	Element	Value
Chromium (Cr)	2915	Rubidium (Rb)	11
Zinc (Zn)	1084	Arsenium (As)	10
Barium (Ba)	380	Cadmium (Cd)	8
Strontium (Sr)	266	Uranium (U)	4
Cupper (Cu)	175	Bromine (Br)	5
Circonio (Zr)	109	Cerium (Ce)	<5
Vanadium (V)	92	Cobalt (Co)	<5
Niobum (Nb)	62	Lanthanum (La)	<5
Lead (Pb)	59	Yttirum (Y)	<3
Nickel (Ni)	26	Thorium (Th)	<3
Tin (Sn)	15	Bismuth (Bi)	<3
Molybdenum (Mo)	11	Gallium (Ga)	<3

### 3.2. Bearing Capacity Tests

**Figure 1:**
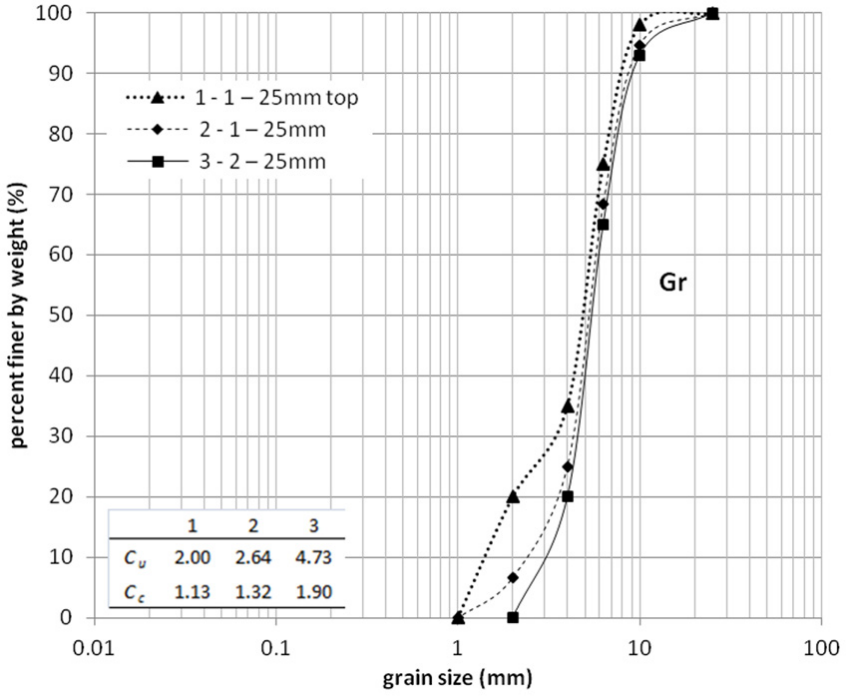
Grain size distribution for steel slag.

Laboratory experimentation of the bearing capacity of steel slag is one of basic tests for classification of unbound material for road structure such as subbase (principal or auxiliary) and subgrade (compacted or natural). For each of those layers, the minimum value of bearing capacity ratio (CBR) is required [36]. It is also a very important parameter used for the design of roads. The results of the tests done on mixture aggregates with grain size range 1–25 mm and 2–25 mm (Figure 1) show that obtained results of CBR are in a range of 35%–42%.

The addition of 1–2 mm fractions to both of the aforementioned mixtures and located in the upper part of samples, allows the obtaining of the bearing capacity ratio on higher levels of more than 60%. The addition was performed by mixing the top layer of the CBR sample with a fraction, whose size was equal to 1–2 mm. Curve 3 (Figure 1) represents sieve analysis results for the top layer. Curve 2 presents the result of sieve analysis for all samples consisting of a mixed top layer. Steel slag was sieved and a gradation curve was estimated. For both mixes, the coefficient of uniformity (*C*u) and coefficient of curvature (*C*c) was calculated. Fractions with the size of 2–25 mm was taken to studies due to possibility of the lack of proper material or the quality control process on the construction area.

**Figure 2:**
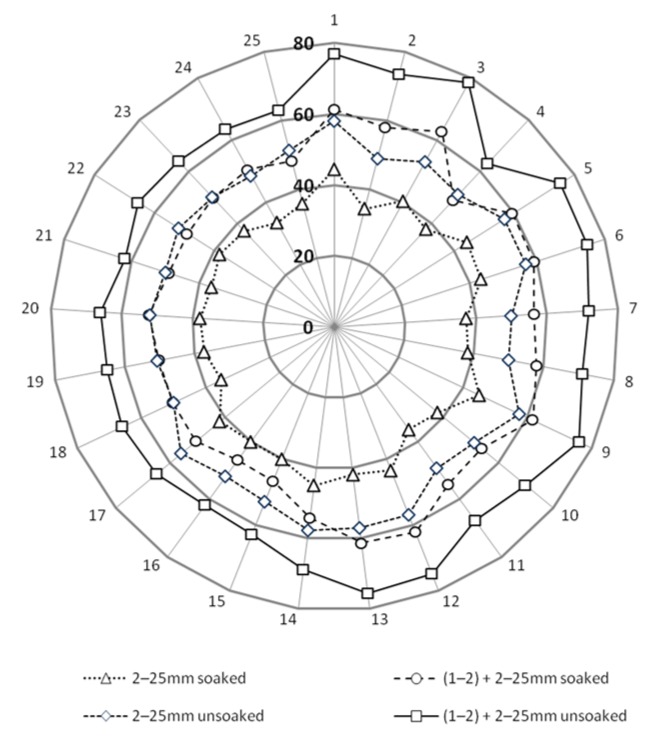
Bearing capacity tests for steel slag mixture aggregate with various grain size and curing conditions.

**Figure 3:**
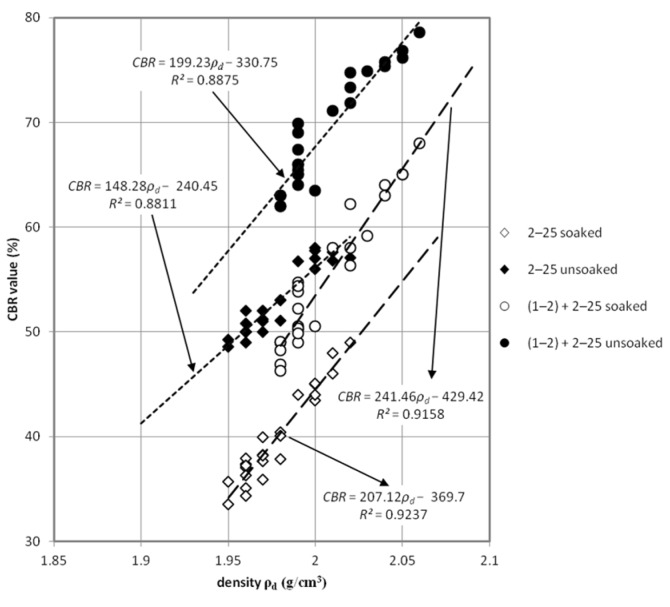
Plot of California bearing ratio (CBR) test results against density of tested samples.

**Figure 4:**
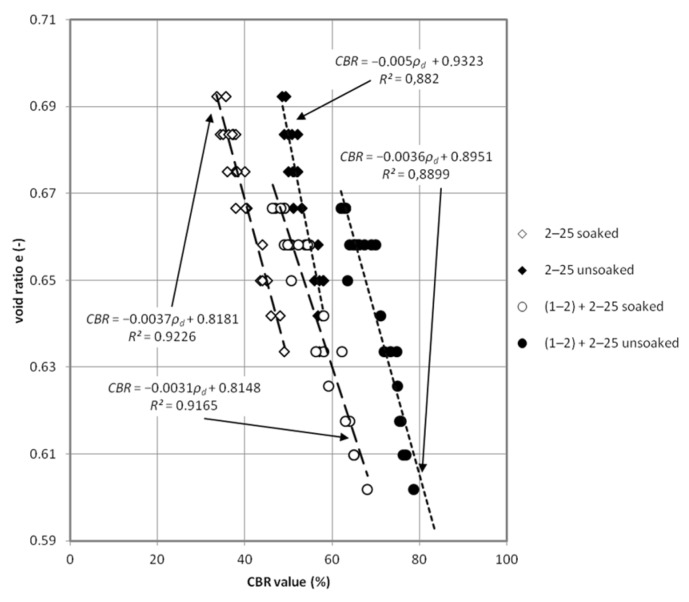
Plot of void ratio diversity for obtained CBR values tested samples.

The received values of CBR allows for the conclusion that the tested material meets the requirements [36] for the subgrade layer of the road (for traffic category KR1-KR6) where the minimum was specified on 35%. Improved by 1–2 mm grain size fraction samples achieved requirements for auxilary subbase (for traffic category KR1-KR6) were 60% CBR values are needed. The quality of CBR results predispose this steel slag material as unbound riding surface where there is no recommendation of minimum value for traffic category KR1-KR2 [36]. Using the analogy with other artificial and recycled (anthropogenic) materials with well-graded curves of grain size distribution in the range of 0–31.5 mm or 0–61 mm, it can be said that slag material is sufficient for principal subbase. Results are presented on Figure 2 where CBR test results are detailed for each test. Figure 3 and Figure 4, present detailed views of CBR values dependence with density and void ratio. Figure 3 presents impact of dry density on CBR value, rise of density has a bigger impact on the mix presented on Curve 3 (Figure 1). In the case of soaked-unsoaked conditions, the difference is also clear. The change of CBR value corresponds with material size grain composition rather than with saturation state. Nevertheless, in both cases, unsoaked samples perform better during the CBR test. Figure 4 presents the impact of void ratio on CBR value. On this plot, the same phenomena can be observed. An increase of CBR value depends on grain size composition. On Figure 3 and Figure 4, the trend of closing interpolated function is also worth noting. This occurrence is caused by the fact that void volume decreasese in the soil skeleton and, because of that, a smaller impact of saturation and bigger contact between particles.

After compaction of this layer, the proposition of adding well graded material (Curve 3) was conducted in studies. These conditions, when two layers of the same material but with other grading would be part of road construction as subbases, for example, were taken into consideration in further studies. Curve 1 (1–25 mm top) is characterized by *C*u 2.00 and *C*c 1.13, which means poor graded material. Curve 3 (2–25 mm) is characterized by *C*u 4.73 and *C*c 1.90, which is stated for well graded material [15]. 

The compaction of gravely materials is problematic and the occurrence of poorly graded material in construction layers could be present. Curve 3 was created on the basis of Curve 1 by adding to 2–25 mm fraction 20% of its weight fraction 1–2 mm. Curve 2 represents average grain size distribution after CBR tests as a control of properly designing the layers in CBR mould (Curve 1 was placed in 1/3 of CBR mould at the top).

**Figure 5:**
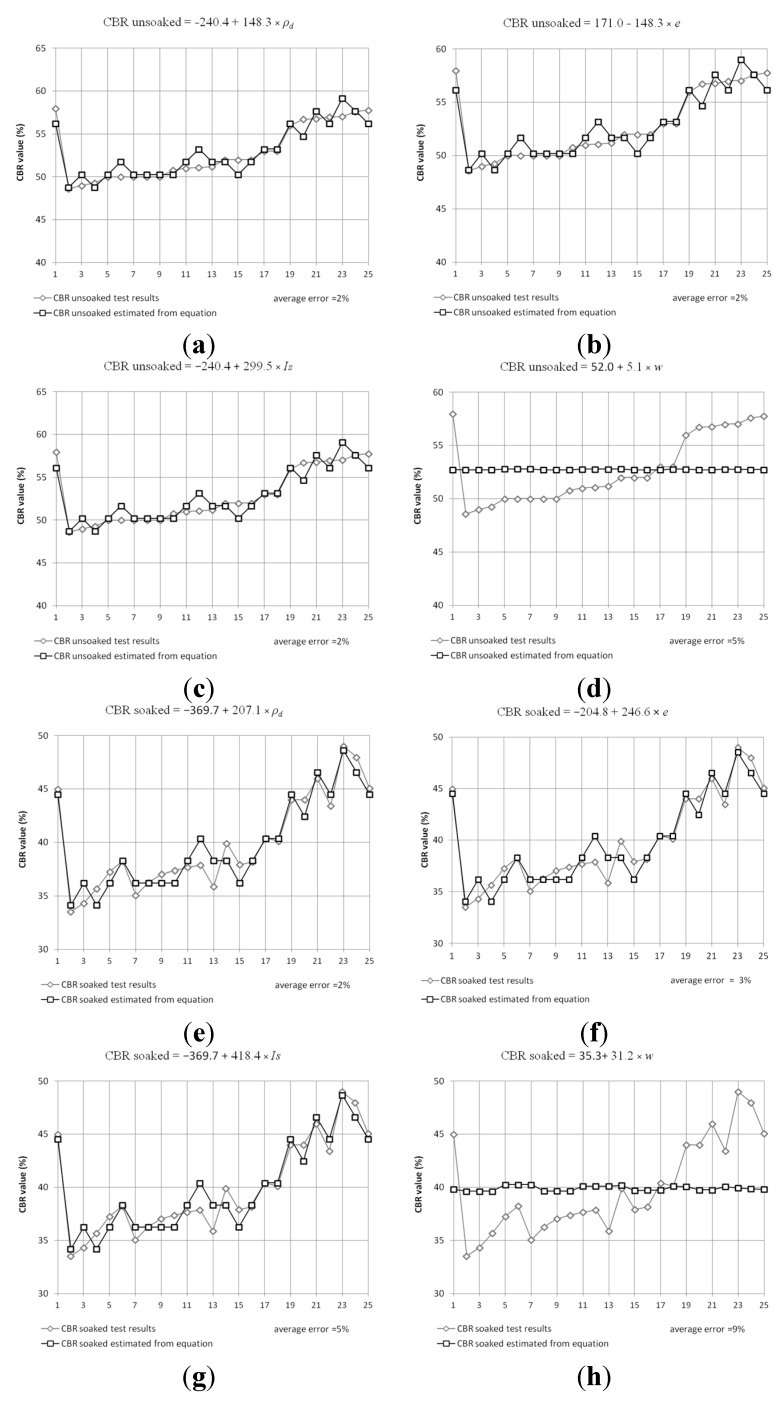
Plot of estimated equations for CBR values and test results for unsoaked test conditions, fraction 2–25 mm. (**a**) CBR unsoaked calculated on the base density. (**b**) CBR unsoaked calculated on the base void ratio. (**c**) CBR unsoaked calculated on the base degree of compaction. (**d**) CBR unsoaked calculated on the base moisture content. (**e**) CBR soaked calculated on the base density. (**f**) CBR soaked calculated on the base void ratio. (**g**) CBR soaked calculated on the base degree of compaction. (**h**) CBR soaked calculated on the base moisture content.

Statistical analysis of obtained test results (Figure 5) was conducted in order to find correlation between physical properties and CBR value of steel slag. Table 3 presents those results. The average error of the presented equation in comparison to test results was between 2% and 5%. 

**Figure 6:**
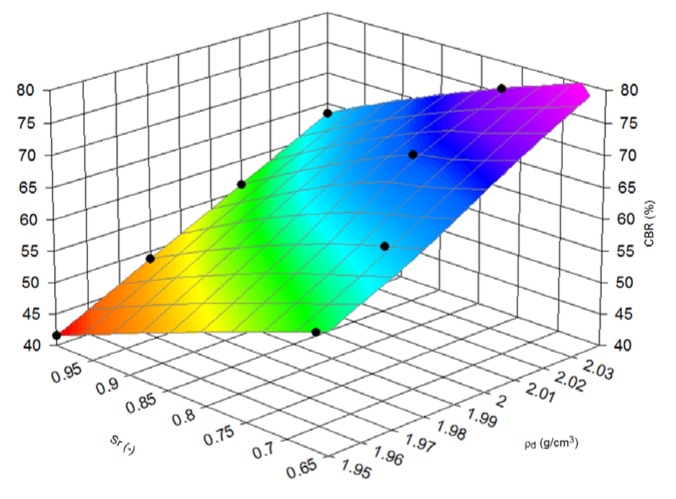
Three-dimensional (3D) view of CBR value dependence from density and saturation ratio (Sr), fraction (1–2) + 2–25 mm.

**Figure 7:**
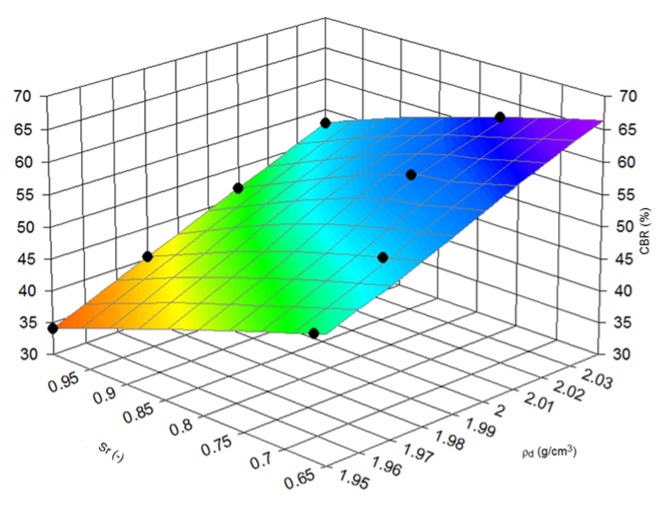
3D view of CBR value dependence from density and saturation ratio, fraction 2–25 mm.

Nevertheless, physical properties calculated with CBR values which have their engineering applications should be extended to more sophisticated relationships. Therefore, the dependence estimation of CBR values and density of samples to degree of saturation during the tests was conducted. This method connects the soaked and unsoaked state of material and clearly, on Figure 6 and Figure 7, presents the decrease of CBR bearing capacity when saturated conditions occur. 

In other words, Figure 6 and Figure 7 concern the impact of saturation. Soaked samples assumed to have 100% moisture and representing the results for soaked and un-soaked specimens with various moisture content were used to find formulas describing this phenomena. 

**Table 3:** Results of statistical correlation estimation from California bearing ratio (CBR) test results. ρ_d_: dry density; *e*: void ratio; *w*: moisture *I*s: relative compaction.

CBR Test Condition	2–25 mm Fraction	(1–2) + 2–25 mm Fraction
CBR unsoaked	*240.4 + 148.3∙*ρ_d_	*330.7 + 139.2∙*ρ_d_
	*171.0 − 176.7∙e*	*227.3 − 245.5∙e*
	*52.0 + 5.1∙w*	*74.3 + 36.8∙w*
	*240.4 + 299.5∙I*_s_	*330.7 + 402.4∙I*_s_
CBR soaked	*369.7 + 207.1∙*ρ_d_	*429.4 + 241.5∙*ρ_d_
	*204.8 − 207.1∙e*	*46.8 − 2297.3∙e*
	*35.3 + 31.2∙w*	*63.6 − 58.6∙w*
	*369.7 + 418.4∙I*_s_	*429.4 + 487.8∙I*_s_

Equation (1) presents a formula for calculating the compressive CBR value (%) of steel slag (*z*) with varying degrees of saturation (-) (*y*) and density (*x*), expressed in g/cm^3^. For fraction (1–2) + 2–25 mm:

(1) $$z=\text{a}+\frac{\text{b}}{x}+\text{c}y+\frac{\text{d}}{x^{2}}+\text{e}y^{2}+\text{f}\frac{x}{y}$$

where letters from a to f are constants: a = 560.1161825; b = −2308.7165; c = 738.5252195; d = 2144.540662; e = −225.272043; f = −803.121929. For this equation the *R*^2^ value is 0.999.

Equation (2) presents a formula for calculating the compressive CBR value of steel slag (%) with varying degree of saturation (-) and density (g/cm^3^), expressed in g/cm^3^. For fraction 2–25 mm:

(2) $$z=\text{a}+\frac{\text{b}}{x}+\text{c}y+\frac{\text{d}}{x^{2}}+\text{e}y^{2}+\text{f}\frac{x}{y}$$

where letters from a to f are constants: a = 124.4164903; b = −880.826622; c = 788.1567559; d = 1064.132286; e = −188.72681; f = −1009.85394. For this equation, the *R*^2^ value is 0.999.

Saturation ratio clearly impacted the results of the CBR tests and the decreasing of bearing capacity from CBR tests although both gradations have the same surface form represented by Equations (1) and (2). Further studies could lead to useful results for engineers’ correlations between soaked and unsoaked conditions. Moreover, when this dependence characterizes the saturation ratio, adding to fraction 2–25 mm, the fraction 1–2 mm, increases the CBR bearing capacity in soaked and unsoaked test conditions.

### 3.3. Cyclic Loading Tests

**Figure 8:**
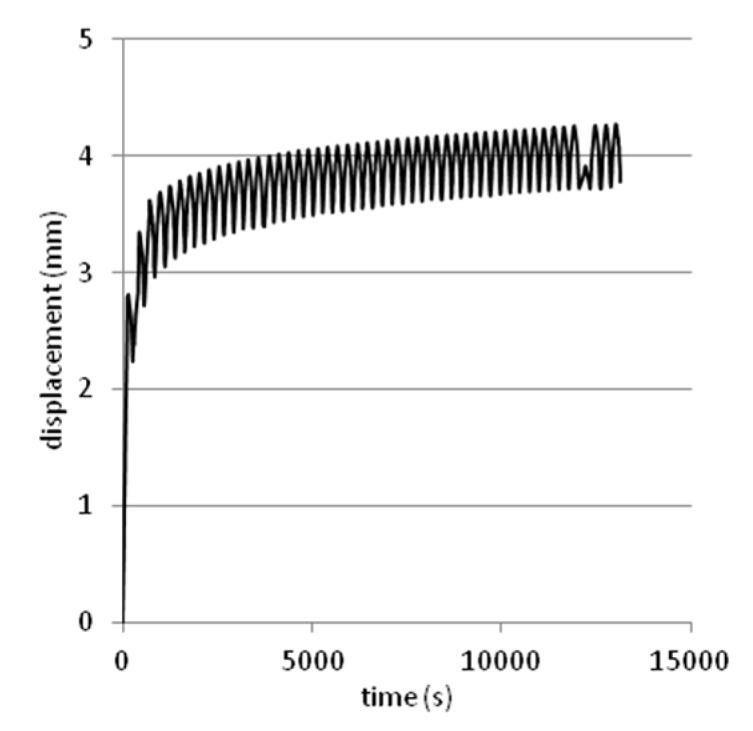
Plot of displacement over time from cCBR tests (soaked conditions).

**Figure 9:**
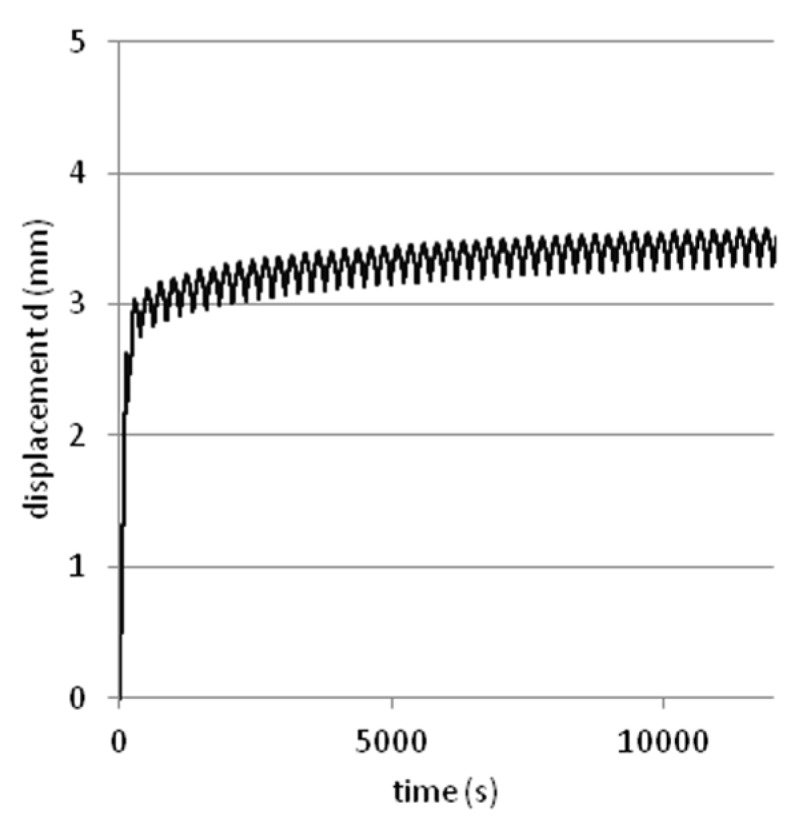
Plot of displacement over time from cCBR tests (unsoaked conditions).

**Figure 10:**
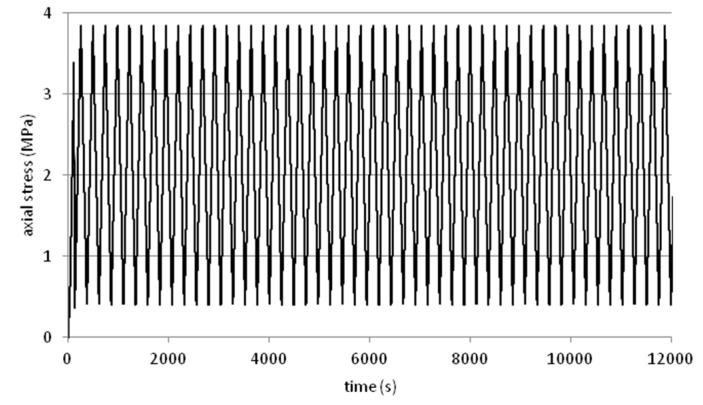
Plot of axial stress over time from cCBR tests (unsoaked conditions).

**Figure 11:**
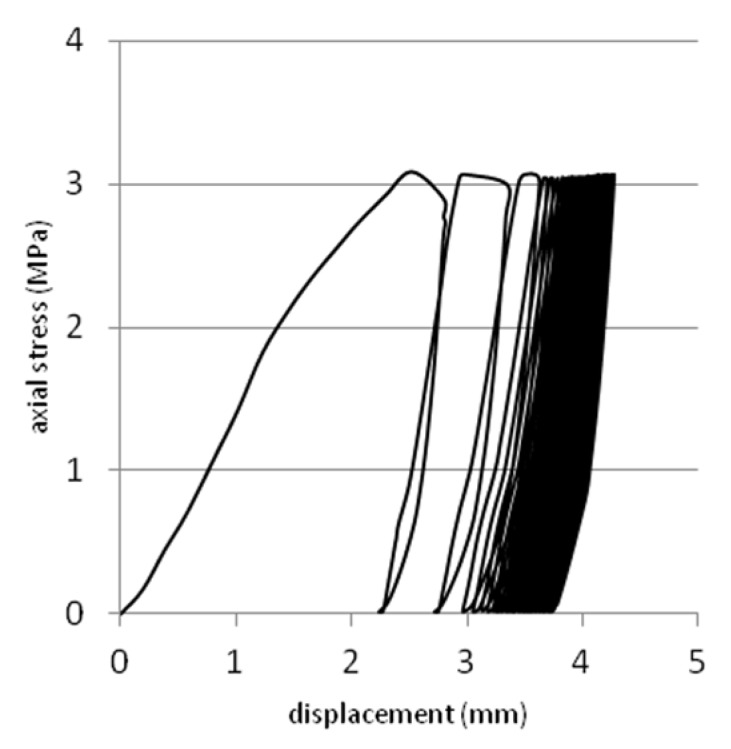
Plot of cCBR test results for steel slag after 50 cycles of loading (soaked conditions).

**Figure 12:**
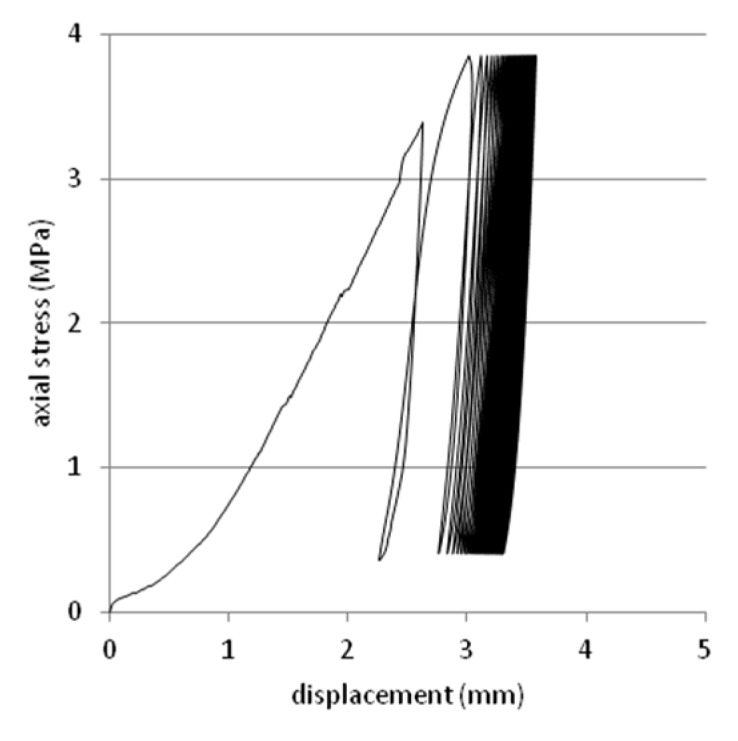
Plot of cCBR test results for steel slag after 50 cycles of loading (unsoaked conditions).

Tests were performed on steel slag that contains 2–25 mm grains and density equal 2.02 g/cm^3^. Results are presented on Figure 8, Figure 9, Figure 10, Figure 11 and Figure 12. Figure 8 and Figure 9 presents a detailed view of displacement variation during loading and unloading phases (soaked and unsoaked conditions, respectively). Figure 10 presents a detailed view of stress variation during the loading and unloading phases. Total displacement after 50 repetitions was equal to 3.57 mm and consists of a 96% elastic response of material to repeated loads. The material was subjected to a stress equal to 3.84 MPa and unloaded to about 10% of maximal stress. The detailed view of this process is presented in Figure 10. Figure 11 and Figure 12 presents views of cCBR tests in axial stress-displacement configuration. 

The resilient modulus cannot be calculated directly from stress-displacement plots and needs to be calculated in another manner. In literature, such recalculation was presented by Arraya [56]. Resilient modulus *M*r from repeated loading CBR can be obtained as follows:

(3) $$M_{\;\text{r}}=\frac{1.513\cdot \left(1-{\text{υ}}^{1.104}\right)\cdot \Delta {\text{σ}}_{\text{p}}\cdot r}{\Delta u^{1.012}}$$

where: ν—Poisson’s ratio (-) (in this study 0.35 for granular materials), Δσ_p_—change between maximum and minimum axial stress in 50th cycle (MPa), *r*—radius of plunger (mm), Δ*u*—recoverable displacement in one cycle (mm).

Resilient modulus for steel slag calculated in this manner is equal to *M*_r_ = 331 MPa which is reasonable result [57].

Figure 8 and Figure 11 presents cCBR tests for fraction 2–25 mm in soaked conditions. Axial stress reached 3.05 MPa and total displacement was 4.26 mm. Important to note is the fact that a huge plastic displacement occurred in the first 3 cycles. It could be explained by delayed pore pressure distribution over the sample and no negative pore pressure in pores which additionally increases the strength of soil mass.

### 3.4. Field Tests

*In situ* static load plate tests (VSS) performed on roads improved by steel slag showed that Young’s modulus (modus of elasticity) *E*_1_ (determined from the first loading of subbase) reached values of 54–72 MPa. The values of Young’s modus (modus of elasticity) *E*_2_ (determined from the second loading of subbase) were 103–126 MPa. The associate soil deformability ratio *I*_0_ reached 1.8–2.0 and fulfilled the requirements *I*_0_ < 2.2. These results show that the tested layer of steel slag fulfilled the requirements for auxiliary subbase (*E*_1_ > 50 MPa).

Static plate load testing done on layers from the second section of road proved that the steel slag mixture fulfilled the requirements for principal subbase ((*E*_1_ > 100 MPa, *E*_2_ > 140–170 MPa) and riding surface. Young’s moduli *E*_1_ were 103–126 MPa and *E*_2_ reached 158–209 MPa. The associate soil deformability ratio *I*_0_ reached 1.5–1.7 and fulfilled the requirements *I*_0_ < 2.2 as well. The value of soil deformability ratio *I*_0_ (*I*_0_ < 2.2) can be also recognized as the compaction ratio I_S_ with value equal to 1.0. It also means that steel slag layers have been well compacted.

**Figure 13:**
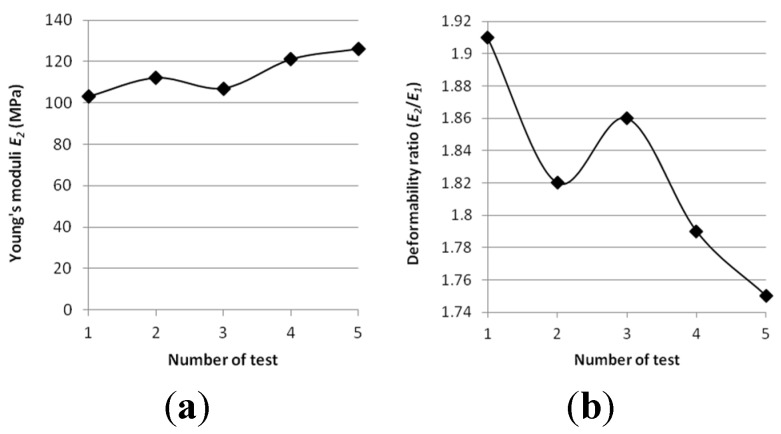
Results of Young’s moduli *E*_2_ and deformability ratio *I*_0_ for ground road improved by steel slag. (**a**) Young’s moduli *versus* number of static plate VSS tests. (**b**) Deformability ratio values *versus* number of static plate VSS tests.

Young modulus plot and Deformability ratio *I*_0_ for each test are presented in Figure 13.

**Figure 14:**
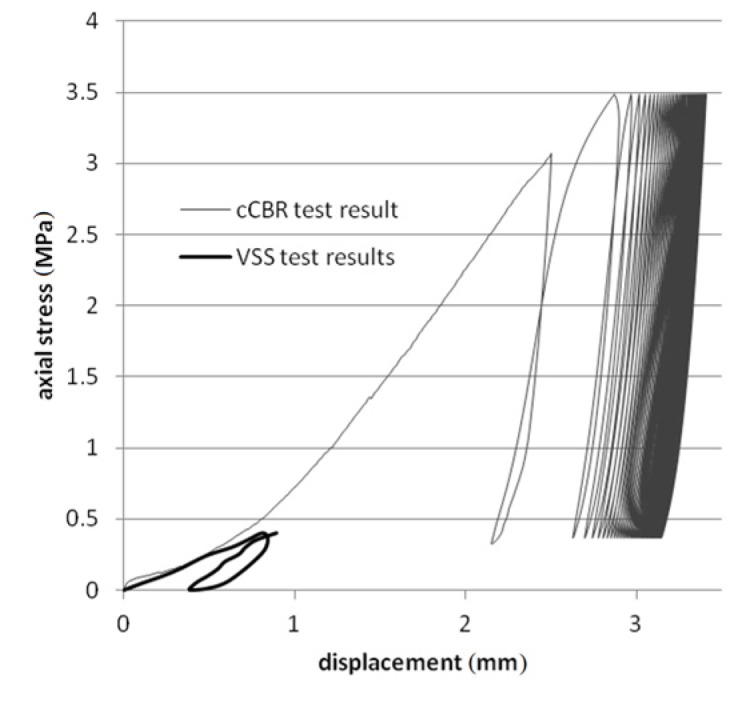
Plot of stress-displacement tests results for cCBR and static load plate VSS on steel slag in duplicate conditions.

Results of cCBR were compared with VSS test data and plotted on a stress-displacement chart (Figure 14). The first loading on a cCBR test overlaps with the VSS test results. Lower displacement obtained during the VSS test leads to greater resilient strain occurrence during the unloading phase. This phenomenon can be utilised to support field studies in laboratories by performing numerous cyclic loading tests and evaluating more reliable parameters for road designers. 

## 4. Conclusions

Aggregates of steel slag have chemical and mechanical properties comparable to similar natural aggregates. An important fact is that varying results are common to virgin soils, so there is no uncertainty regarding performance under working stress. However, due to their heterogeneity, it is important to verify them before using. 

Their parameters fill requests for road construction independently from their origin. The designated high content of chromium and zinc are strongly associated with the internal crystal structure of steel slag. Consequently, they can be safely applied in roads’ structure. 

The aforementioned results suggest a possibility to recycle steel slag as a material for subbases. Test results concerning lack of fines and soaked conditions were mixed. Those circumstances are negative and mostly affect the performance of subgrade constructed from steel slag. CBR tests show a drop of bearing capacity of this material when the aforementioned phenomena occur. Therefore, the constructed subbase is cut off from any water inflows or outflows. This treatment makes obtaining steady conditions possible. Well-graded steel slag reached the CBR value exceeding 60% in each of the 25 repetitions. On the other hand, poorly graded soaked samples mostly did not cross 40% of CBR value. Upon comparing the impact of the previously mentioned properties on bearing capacity gradation and saturation conditions, steel slag is more sensitive to poor gradations or simply to a lack of fine graded grains. 

Saturation ratio decreases bearing capacity from CBR tests. Equations (1) and (2) could in further studies lead to the estimation of a new equation, which can take into account change in gradation. This is made possible by the fact that for steel slag with varying gradation, a change of CBR value with density and saturation ratios has the same surface represented by Equations (1) and (2). 

Moreover, cyclic loading shows good performance of steel slag and plastic displacement was 1 mm greater after the 50th load repetition than after the first loading. Eurocode 7 EN 13286-7:2004 [58] classifies steel slag by its mechanical performance by the resilient modulus *M*_r_ parameter, and this material reached the C2 class. 

Field tests including the static plate load test have proved that steel slag mixture fulfilled the requirements for principal subbase ((*E*_1_ > 100 MPa, *E*_2_ > 140–170 MPa) and riding surface. It is interesting to note that VSS tests and cCBR tests seem to be interrelated. This would be important for supporting field tests with laboratory studies. Mechanistic-empirical pavement design states that, for every layer designed, the resilient modulus *M*_r_ should be taken in the calculation. The cCBR, therefore, could be a response to the lack of simple methods for estimating such a parameter. 

Taking into consideration the above test results, it can be concluded that steel slag can be used in base courses in road structures for motorways and roads with medium traffic loads. The most important selected geotechnical parameters, bearing capacity ratio (obtained from laboratory tests) and resilient modulus *M*_r_ showed that their values correspond with requirements for subbase (principal or auxiliary) and riding surface as well. The use of steel slag in road structure courses would be desired from both the economic and environmental point of view: great quantities of waste material would thus be used, reducing the amount of slag deposited in landfills.
